# Okuläre Manifestationen bei COVID-19-Patienten

**DOI:** 10.1007/s00347-022-01581-y

**Published:** 2022-01-28

**Authors:** Kristin Hösel, Claus von der Burchard, Domagoj Schunk, Jeanette Franzenburg, Thomas Bahmer, Derk Frank, Justina Dargvainiene, Johann B. Roider

**Affiliations:** 1 Klinik für Augenheilkunde, UKSH Kiel, Arnold-Heller-Str. 3, 24104 Kiel, Deutschland; 2 Department of Internal Medicine, UKSH Kiel, Kiel, Deutschland; 3 Department of Clinical Chemistry, UKSH Kiel, Kiel, Deutschland; 4 DZHK (German Centre for Cardiovascular Research), Kiel, Deutschland

**Keywords:** SARS-CoV-2, Retinale Manifestationen, Vaskuläre Fundusveränderungen, Übertragung über Tränenflüssigkeit, Virusprävalenz, SARS-CoV-2, Retinal manifestations, Vascular alterations, Transmission via tears, Viral shedding

## Abstract

**Hintergrund:**

Insbesondere okuläre Manifestationen bei COVID-19 wurden bisher kaum in prospektiven Studien erfasst.

**Ziel der Arbeit:**

Das Ziel dieser Studie war es, die COVID-19-assoziierten Veränderungen des vorderen und hinteren Augenabschnittes zu evaluieren sowie die Prävalenz von SARS-CoV-2-RNA in der Tränenflüssigkeit zu analysieren.

**Methoden:**

Patienten mit positivem Nasen-Rachen-Abstrich, welche sich vom 16.04.2020 bis 07.01.2021 stationär im Universitätsklinikum Schleswig-Holstein, Campus Kiel, befanden, wurden eingeschlossen. Der vordere Augenabschnitt sowie der hintere Augenabschnitt in Mydriasis wurden augenärztlich untersucht. Von beiden Augen wurde zusätzlich ein Schirmer-Tränenstreifen auf SARS-CoV-2-RNA analysiert.

**Ergebnisse:**

Es wurden 37 Patienten eingeschlossen. Am vorderen Augenabschnitt zeigten sich Chemosis (5), Hyposphagma (2) und Konjunktivitis (1). Bei 11 Patienten zeigten sich vaskuläre Alterationen und möglicherweise krankheitsspezifische Manifestationen am Augenhintergrund in einem oder beiden Augen: retinale Blutungen (5), Cotton-Wool-Spots (5) und Tortuositas (5). Bei einem Patienten fand sich ein Arterienastverschluss, bei einem anderen Patienten ein Venenastverschluss. Zwei Patienten hatten einen positiven Bindehautabstrich in einem oder beiden Augen.

**Diskussion:**

Die in dieser Studie beschriebenen Veränderungen des vorderen Augenabschnittes sind in einer Vielzahl chinesischer Studien vorbeschrieben, jedoch nicht spezifisch für COVID-19. Zudem konnten diverse vaskuläre Funduspathologien gefunden werden, welche sich signifikant häufiger bei COVID-19-Patienten zeigten als bei einer gesunden Vergleichskohorte. Es bleibt unklar, ob diese Veränderungen direkt durch SARS-CoV‑2 ausgelöst werden oder ob sie auf systemischen Komorbiditäten basieren. SARS-CoV-2-Virusprävalenz in Tränenflüssigkeiten ist möglich.

## Abstract (en)

**Background:**

There are only few reports on ocular symptoms and manifestations in association with coronavirus disease 2019 (COVID-19).

**Objective:**

The aim of this study is to describe ocular manifestations in the anterior and posterior segments of the eye and to analyze viral prevalence in tears of patients with COVID-19.

**Methods:**

Hospitalized COVID-19 patients treated from 16 April 2020 to 7 January 2021 at this hospital were screened for ocular manifestations in the anterior and posterior segments. Conjunctival swabs were analyzed for severe acute respiratory syndrome coronavirus 2 (SARS-CoV-2) RNA.

**Results:**

A total of 37 patients were enrolled in this study. In the anterior segment we found chemosis of the conjunctiva (5), hyposphagma (2) and conjunctivitis (1). In 11 patients vascular alterations and potentially disease-specific manifestations of the fundus were found in one or both eyes: retinal hemorrhages (5), cotton wool spots (5) and tortuosity (5). One patient demonstrated branch artery occlusion, one had branch retinal vein occlusion and two patients had positive conjunctival swab results in one or both eyes.

**Conclusion:**

Our findings of the anterior segment are commonly known, although not specific for COVID-19. Various vascular fundus abnormalities were found in the study; however, it is unclear whether these were correlated to systemic comorbidities or whether they were caused or exacerbated by COVID-19. This study suggests that the risk of viral transmission via tears is low.

Seit dem Beginn der Pandemie durch das infektiöse Atemwegssyndrom-Coronavirus Typ 2 (SARS-CoV-2) Virus gibt es mehr als 200 Mio. Infizierte weltweit. Schnell wurde deutlich, dass es sich bei der Infektionskrankheit Coronavirus-Krankheit-2019 (COVID-19) nicht um eine reine Atemwegserkrankung handelt, sondern vielmehr jedes Organ betroffen sein kann [1]. Dennoch gibt es nur wenige Erkenntnisse zu okulären Manifestationen.

## Hintergrund und Fragestellung

Sei dem tragischen Tod des Ophthalmologen Dr. Li Wenliang gibt es eine Reihe von Überlegungen zu Transmissionswegen des Virus sowie möglichen Präventionsmöglichkeiten [2, 3]. Frühe Studien, v. a. aus China, belegen die Möglichkeit der viralen Ausbreitung über Tränenflüssigkeit [4–6]. Zur grundlegenden Analyse von okulären Manifestationen gibt es jedoch nur wenige prospektive Studien [7]. Insbesondere zu Veränderungen am Augenhintergrund fehlen umfangreiche Studien trotz zahlreicher Fallberichte zu Gefäßverschlüssen und vaskulären Veränderungen [8–10].

Mit zunehmendem Wissen über pathogenetische und immunologische Mechanismen von COVID-19 sind Fundusveränderungen durch Endotheldysfunktion, Gefäßentzündung, direkte virale Schädigung oder durch das Virus induzierte Immunschwächen denkbar [11]. Casagrande et al. fanden SARS-CoV-2-RNA in retinalen Biopsien von an COVID-19 Verstorbenen [13]. In der klinischen Untersuchung lassen diese sich jedoch schwierig von Fundusveränderungen durch gefäßassoziierte Erkrankungen wie Diabetes mellitus, arteriellem Hypertonus oder Endokarditis [12] abgrenzen.

Die SERPICO-19-Studie (Italien) versucht daher den Vergleich mit einer repräsentativen Kontrollgruppe. Es handelt sich um eine Querschnittstudie, in welcher Fundusfotografien von insgesamt 54 COVID-19-Patienten mit denen von 133 nicht exponierten Probanden verglichen wurden. In den Fundusbildern der erkrankten Patienten fanden sich signifikant häufiger Netzhautblutungen, Cotton-Wool-Spots, dilatierte Venen und Tortuositas. Des Weiteren konnten die Autoren dieser Studie zeigen, dass der Gefäßdurchmesser retinaler Venen signifikant größer bei an COVID-19 Erkrankten war als bei gesunden Probanden. Die Gefäßgröße korrelierte dabei auch mit dem Schweregrad der Erkrankung [14].

In einer anderen prospektiven Beobachtungsstudie an 43 COVID-19-Patienten wurden bis auf eine einseitige Chorioretinitis keine weiteren retinalen Manifestationen gefunden [15].

Das Ziel dieser Studie war es, prospektiv okuläre Manifestationen bei COVID-19 am vorderen und hinteren Augenabschnitt zu beschreiben sowie die Tränenflüssigkeit auf Virus-RNA zu analysieren.

## Studiendesign und Untersuchungsmethoden

Es handelt sich um eine prospektive (nichtinterventionelle) Beobachtungsstudie. Für die Studie wurden Patienten mit einem positiven nasopharyngealen PCR-Abstrich rekrutiert, welche sich im Zeitraum vom 16.04.2020 bis 07.01.2021 stationär im Universitätsklinikum UKSH Kiel befanden. Von allen ansprechbaren Patienten wurde eine Einverständniserklärung eingeholt. Bei Patienten, welche aufgrund ihres Krankheitszustandes nicht einwilligungsfähig waren, wurde von einer mutmaßlichen Einwilligung zur Studie ausgegangen. Die Zustimmung durch die lokale Ethikkommission der Christian-Albrechts-Universität zu Kiel lag vor Beginn vor (Protokoll Nr. D 469/20).

Klinische Daten wie Alter, Geschlecht, Zeitpunkt der Hospitalisation und Abstrichergebnisse wurden aus der elektronischen Krankenakte übernommen. Ansprechbare Patienten wurden nach allgemeinen und okulären Symptomen befragt. Die klinischen Untersuchungen wurden von einer Weiterbildungsassistentin für Augenheilkunde durchgeführt (KH). Dabei wurde auf die Einhaltung der gängigen Infektionsschutzmaßnahmen geachtet, und stets wurden Einwegschutzkittel, -handschuhe, FFP2-Maske sowie Schutzbrille getragen. Der vordere Augenabschnitt wurde mit einer Handspaltlampe auf das Vorhandensein von Konjunktivitis, Skleritis, Chemosis, Hyposphagma, Karunkelschwellung, Membranbildung oder andere Pathologien untersucht. Für die Untersuchung des hinteren Augenabschnittes erfolgte die Pupillenerweiterung mit Tropicamid-Augentropfen. Es wurde nach vaskulären und infektiösen retinalen Alterationen gescreent wie Gefäßveränderungen, Netzhautblutungen, Cotton-Wool-Spots und Infiltrate. Von beiden Augen wurde von allen Patienten zusätzlich Tränenflüssigkeit mittels Schirmer-Tränenstreifens gewonnen und auf SARS-CoV-2-RNA analysiert. Hierfür wurden die Schirmer-Streifen für ca. 30 s in den unteren Bindehautsack eingelegt. Die weitere Aufarbeitung erfolgte im Labor. Die Schirmer-Streifen wurden in 700 µl in Copan Universal Transport Medium (UTM®, COPAN Diagnostics, CA, USA) inkubiert und das Homogenat mit AltoStar® Purification Kit 1.5 (altona Diagnostics, Hamburg) oder Mag-Bind® Viral DNA/RNA 96 Kit (Omega, GA, USA) extrahiert. Für die diagnostischen RT-PCRs wurde das RealStar® SARS-CoV‑2 RT-PCR Kit RUO (altona Diagnostics, Hamburg) mit dual target system (E and S Gen) benutzt. Die Analyse erfolgte mit dem CFX96™ Deep Well Real-Time Detection System (IVD, Bio-Rad, Feldkirchen) und der CFX Manager Software (IVD, Bio-RAD, Feldkirchen). Die Nachweisgrenze liegt laut Herstellerangaben bei 1,00E-01 PFU/ml (1,000 copies/ml).

Die Fundusveränderungen wurden mit der gesunden Vergleichskohorte aus der SERPICO-19-Studie verglichen. Dabei wurde der Exakte Fisher-Test angewandt.

## Ergebnisse

Klinische Daten, okuläre Symptome und Manifestationen sowie die Ergebnisse der Tränenproben sind in Tab. 1 aufgelistet.

**Table 1:** 

	Anzahl Patienten (%), gesamt = 37
*Mittleres Alter in Jahren (Range)*	60,5 (22–93)
*Geschlecht*	
Männlich	25 (67,6)
Weiblich	12 (32,4)
*Allgemeine COVID-19-Symptome*	29 (78,3)
*Intubiert und beatmet*	8 (21,6)
*Okuläre Symptome*	
Epiphora	1 (2,7)
Sehverschlechterung	2 (5,4)
*Pathologien des vorderen Augenabschnittes*	
Konjunktivitis	1 (2,7)
Chemosis	5 (13,5)
Hyposphagma	2 (5,4)
Skleritis	0
Karunkelschwellung	0
Membranbildung	0
*Pathologien des hinteren Augenabschnittes*	
Netzhautblutungen	5 (13,5)
Cotton-Wool-Spots	5 (13,5)
Tortuositas	5 (13,5)
Venöser Verschluss	1 (2,7)
Arterieller Verschluss	1 (2,7)
*Schirmer-Tränenstreifen (RT-PCR)*	
Positiv	2^a^ (5,4)
Negativ	35^b^ (94,6)

^a^Bei 2 Patienten in insgesamt 3 Augen konnte SARS-CoV‑2 in Tränenflüssigkeit nachgewiesen werden

^b^Bei 35 Patienten zeigte sich an beiden Augen kein SARS-CoV-2-Nachweis im Schirmer-Streifen. Insgesamt waren 71 Schirmer-Proben negativ

In dem angegebenen Zeitraum wurden 37 Patienten untersucht. Das mittlere Alter betrug 60,5 Jahre (22–93, Standardabweichung ±18,7). Alle Patienten hatten einen im stationären Aufenthalt bestätigten positiven Nasen-Rachen-Abstrich auf SARS-CoV-2-RNA. Die ophthalmologische Untersuchung fand im Mittel 4,4 Tage nach dem positiven Abstrichergebnis (Standardabweichung ±2,7) und 7,6 Tage nach dem Auftreten der ersten klinischen COVID-19-Symptome statt (Standardabweichung ±7,5). In 23 Fällen (62,2 %) lag der Studieneinschluss innerhalb von 3 Tagen nach positivem Nasen-Rachen-Abstrich. Bei 8 der 37 Patienten handelte es sich beim positiven Nasen-Rachen-Abstrich um einen Zufallsbefund, welcher im Rahmen eines routinemäßigen Screenings durchgeführt wurde, z. B. bei stationärer Aufnahme aus anderen Gründen. Diese Patienten gaben im gesamten stationären Verlauf keine allgemeinen COVID-19-Symptome an trotz bestätigtem PCR-Nachweis im Nasen-Rachen-Abstrich. Sie blieben auf der Isolierstation bis zum stationären Behandlungsende, welches nach Ermessen der jeweiligen Fachrichtung festgelegt wurde, jedoch mindestens 3 Tage.

Es konnten 28 der 37 Patienten die Frage nach allgemeinen oder okulären Symptomen beantworten. Neun Patienten waren nicht ansprechbar, da sie beatmet (8) oder in schlechtem Allgemeinzustand (1) waren. Am Untersuchungstag litten 29 der 37 Patienten an allgemeinen COVID-19-Symptomen wie Fieber, Atemnot, Husten, Geruchs- oder Geschmacksstörungen oder waren beatmet; 25 der 28 ansprechbaren Patienten verneinten okuläre Symptome. Zwei Patienten gaben akute Sehstörungen auf einem Auge an. Ein Patient berichtete über vermehrtes Tränenträufeln auf beiden Augen.

In der Spaltlampenuntersuchung fanden sich Konjunktivitis (1), Chemosis der Bindehaut an mindestens einem Auge (5) sowie Hyposphagma (2) an mindestens einem Auge. Die Konjunktivitis zeigte sich im selben Patienten, welcher auch vermehrtes Tränenträufeln angegeben hatte. Alle 7 Patienten mit entweder Chemosis oder Hyposphagma waren beatmete Patienten. Skleritis, Karunkelschwellung oder Membranbildung fanden sich bei keinem der Patienten; 29 von 37 Patienten wiesen keine infektassoziierten Pathologien des vorderen Augenabschnittes auf.

Bei insgesamt 11 Patienten zeigten sich vaskuläre und somit potenziell erkrankungsspezifische Fundusveränderungen. Es gab hierbei keinen signifikanten Unterschied zwischen beatmeten und nicht beatmeten Patienten (*p* = 0,2003, Fisher-Test). Es zeigten sich retinale Hämorrhagien bei 5 Patienten, Cotton-Wool-Spots bei 5 Patienten, Tortuositas bei 5 Patienten, ein Arterienastverschluss bei einem Patienten und ein Venenastverschluss bei einem Patienten; 26 der 37 Patienten wiesen keine COVID-19-assoziierten Fundusveränderungen auf.

Es wurde von allen 37 Patienten je Auge ein Schirmer-Tränenstreifen eingesandt. In 71 der 74 Tränenproben konnte keine SARS-CoV-2-RNA nachgewiesen werden. Ein Patient hatte an beiden Augen einen positiven Tränennachweis. Dieser Patient zeigte auch eine einseitige Venenastthrombose. Bei diesem Patienten gab es keine Pathologien oder Irritationen des vorderen Augenabschnittes. Ein anderer Patient hatte einen positiven Tränennachweis auf dem rechten Auge. Dieser Patient war beatmet und hatte ebenfalls eine beidseitige Chemosis der Konjunktiva.

## Diskussion

Spaltlampenuntersuchung und Fundoskopie sind schnelle, günstige und nichtinvasive Methoden, um mögliche infektiöse und vaskuläre okuläre Pathologien am Patienten zu erkennen. In dieser Studie beschreiben wir okuläre Befunde bei COVID-19-Patienten und analysieren den viralen SARS-CoV-2-Nachweis in Tränenflüssigkeit.

In unserer Studie lag die ophthalmologische Untersuchung im Mittel 7,6 Tage nach Auftreten der ersten COVID-19-Symptome. Bei mindestens 24 von 37 Patienten lag die Untersuchung innerhalb des Krankheitsintervalls von 2 Wochen, in welchem in der Regel auch Viruslast im Nasen-Rachen-Abstrich gefunden werden kann [16] (ohne Berücksichtigung der 8 Patienten, welche im gesamten stationären Aufenthalt keine Krankheitssymptome entwickelten).

Von 37 COVID-19-Patienten zeigte 1 Patient Konjunktivitis, 5 Patienten zeigten Chemosis der Konjunktiva und 2 Patienten Hyposphagma an mindestens einem Auge. Alle Patienten mit Chemosis und Hyposphagma waren beatmet. Aufgrund dieser hohen Korrelation mit dem Beatmungsstatus sind Chemosis und Hyposphagma möglicherweise als Nebeneffekt von Intubation oder Beatmung zu sehen. Ähnliche Beobachtungen am vorderen Augenabschnitt bei COVID-19-Patienten sind auch in anderen Studien beschrieben [4–6].

Als mögliche retinale Manifestationen zeigten sich Netzhautblutungen, Cotton-Wool-Spots, Tortuositas sowie retinale Gefäßverschlüsse. Marinho et al. fanden ähnliche Beobachtungen bei 4 von 12 Patienten [17]. Auch in der SERPICO-19-Studie zeigten sich neben signifikant vergrößerten Gefäßdurchmessern vermehrt vaskuläre Funduspathologien wie retinale Blutungen, Cotton-Wool-Spots und Tortuositas. In der unexponierten gesunden Vergleichskohorte aus 133 gesunden Probanden fanden sich nur in einer unsignifikanten Minderheit retinale Alterationen [14]. Um unsere Ergebnisse zu beurteilen, verglichen wir unsere Beobachtungen mit den veröffentlichten Daten dieser Vergleichskohorte (Tab. 2). Es fanden sich signifikant mehr vaskuläre Pathologien unter den COVID-19-Patienten als bei der gesunden Kontrollgruppe. Dies legt nahe, dass COVID-19 mit vaskulären retinalen Manifestationen assoziiert sein kann. Während in der SERPICO-Studie die erweiterte Einteilung in schwer erkrankte („severe“) und nicht schwer erkrankte („non-severe“) COVID-Patienten erfolgte, um eine positive Korrelation der Vergrößerung der Gefäßdurchmesser mit der Schwere der Erkrankung aufzuzeigen, wurde in unserer Studie keine Korrelationsanalyse mit der Schwere der Erkrankung durchgeführt. Jedoch zeigte sich in unserer Studie kein signifikanter Unterschied zwischen den beatmeten und nicht beatmeten COVID-19-Patienten.

**Table 2:** 

	COVID-19-Patienten, *n* = 37	Gesunde Probanden der SERPICO-Studie, *n* = 133	*p*-Value^a^
Netzhautblutungen (%)	5 (13,5)	2 (1,5)	*p* = 0,006
Cotton-Wool-Spots (%)	5 (13,5)	0	*p* = 0,0004
Tortuositas (%)	5 (13,5)	9 (6,7)	*p* = 0,19
Venöser Verschluss (%)	1	0	*p* = 0,22
Arterieller Verschluss (%)	1	0	*p* = 0,22

^a^Fisher exact test for qualitative variables

Es gibt mehrere Fallberichte von retinalen Verschlüssen im Zusammenhang mit COVID-19, welche sowohl venöse [8, 18] als auch arterielle Gefäße [19, 20] betreffen. Auch wenn die Pathomechanismen noch nicht vollständig geklärt sind, scheinen hyperinflammatorische Prozesse die Inzidenz von arteriellen oder venösen thrombembolischen Ereignissen zu erhöhen. In fast allen Organsystemen wurden vaskuläre Begleiterscheinungen bei COVID-19 gefunden [21].

In unserer Studie zeigte sich bei 35 von 37 Patienten keine SARS-CoV-2-Viruslast in der Tränenflüssigkeit trotz positivem Nasen-Rachen-Abstrich. Die Prävalenz eines positiven Nachweises in Tränenflüssigkeit liegt in unserer Studie bei 4,1 % und stimmt mit den Prävalenzen aus anderen Studien überein (3,3–5,8 %) [5, 22]. Die niedrige Prävalenz zeigt, dass die Infektion via Tränenflüssigkeit möglich, jedoch insgesamt eher unwahrscheinlich ist. Erwähnenswert ist, dass in einem Fall die Tränenflüssigkeit positiv war, obwohl der Patient erst Tage später allgemeine klinische Symptome entwickelte.

Unsere Studie hatte einzelne Limitationen: Erstens konnten wir nur eine relativ geringe Patientenzahl einschließen. Das liegt größtenteils in der geringen Zahl an stationären COVID-19-Patienten in Norddeutschland im Untersuchungszeitraum begründet. Laut Robert Koch-Institut gab es im Rekrutierungszeitraum (16.04.2020 bis 07.01.2021) etwa 31.000 registrierte SARS-CoV-2-Infektionen in Schleswig-Holstein [23]. Da der Großteil der COVID-19-Patienten nur marginale respiratorische Symptome aufweist, war eine stationäre Behandlung glücklicherweise nur selten notwendig.

Zweitens ist das Design unserer Studie eher eine beobachtende Fallanalyse ohne Kontrollgruppe mit gesunden Probanden. Vaskuläre Alterationen wie Netzhautblutungen, Cotton-Wool-Spots und Tortuositas sind auch klinische Zeichen anderer Erkrankungen. Es bleibt unklar, ob diese Pathologien eher mit systemischen Komorbiditäten oder der COVID-19-Erkrankung assoziiert sind. Wir führten keine Korrelationsanalyse durch, um den Zusammenhang dieser Veränderungen mit chronischen Grunderkrankungen wie Diabetes mellitus, arterielle Hypertonie usw. zu untersuchen. Es ist ebenso möglich, dass inflammatorische Reaktionen durch das Virus oder auch die Behandlung der Erkrankung stärkere Auswirkungen auf Patienten mit chronischen vaskulären Grunderkrankungen haben als auf vorher gesunde Patienten. Vaskuläre Komplikationen sind auch nach COVID-19-Impfungen beschrieben. Der Impfstoff-assoziierten Thrombozytopenie („vaccine-induced immune thrombotic thrombocytopenia“ [VITT]) liegt wahrscheinlich eine Autoimmunreaktion auf COVID-19-Impfstoffe zugrunde, welche zu Thrombozytopenien und Thrombosen führen kann. Der genaue Pathomechanismus dieser seltenen Komplikation ist bisher unbekannt, ebenso wie die Bedeutung im Hinblick auf mögliche retinale Gefäßverschlüsse nach der Impfung [24].

Drittens hat sich die Schirmer-Teststreifenmethode zwar als erfolgreiche Nachweismethode viraler Konjunktivitiden herausgestellt, jedoch ist die Sensitivität des SARS-CoV-2-Nachweises in Tränenflüssigkeit unklar. Auch bleibt offen, ob und mit welcher Wahrscheinlichkeit das Virus über die Bindehaut aufgenommen und zu weiteren okulären Manifestationen führen kann. SARS-CoV‑2 bindet an den Angiotensin-konvertierendes Enzym 2(ACE-2)-Rezeptor, um intrazellulär aufgenommen zu werden [25]. Das Vorhandensein von ACE-2-Rezeptoren in der Bindehaut wird in der Literatur jedoch kontrovers diskutiert [26, 27]. Dennoch scheint der Ausbreitungsweg per continuitatem von der Bindehaut in den hinteren Augenabschnitt möglich [28].

Unsere Ergebnisse zeigen, dass das Risiko einer COVID-19-Manifestation im vorderen Augenabschnitt, insbesondere einer konjunktivalen Infektion oder einer Transmission über die Tränenflüssigkeit, als gering einzuschätzen ist. Aus unserer Sicht besteht das größte Infektionsrisiko für OphthalmologInnen sowie medizinisches Personal in der Transmission via Aerosole und Tröpfchen [29].

Unsere Beobachtungen weisen darauf hin, dass COVID-19 zu gefäßassoziierten Fundusveränderungen führen kann. In Zukunft muss der Zusammenhang mit vaskulären Komorbiditäten grundlegend untersucht werden. Auch fehlen Langzeitdaten zu späten okulären Komplikationen nach COVID-19-Erkrankung.

## Fazit für die Praxis

- COVID-19-Manifestationen am vorderen Augenabschnitt sind unspezifisch.
- Retinale COVID-19-Manifestationen beschreiben vaskuläre Alterationen wie retinale Blutungen, Cotton-Wool-Spots, Tortuositas und Gefäßverschlüsse.
- SARS-CoV-2-Virusnachweis in Tränenflüssigkeit ist möglich, jedoch selten.

## Abkürzungen

ACE‑2: Angiotensin-konvertierendes Enzym 2
COVID-19: Coronavirus disease 2019 (deutsch Coronavirus-Krankheit-2019)
RNA: Ribonucleic acid (deutsch Ribonukleinsäure)
RT-PCR: Reverse transcriptase-polymerase chain reaction (deutsch Reverse-Transkriptase-Polymerasekettenreaktion)
SARS-CoV‑2: Severe acute respiratory syndrome coronavirus 2 (deutsch schweres akutes Atemwegssyndrom-Coronavirus Typ 2)

## References

[1] Zaim S, Chong JH, Sankaranarayanan V (2020). COVID-19 and multiorgan response. Curr Probl Cardiol.

[2] Parrish RK, Stewart MW, Duncan Powers SL (2020). Ophthalmologists are more than eye doctors—in memoriam Li Wenliang. Am J Ophthalmol.

[3] Sommer A (2020). Humans, viruses, and the eye—an early report from the COVID-19 front line. JAMA Ophthalmol.

[4] Seah IYJ, Anderson DE, Kang AEZ (2020). Assessing viral shedding and Infectivity of tears in Coronavirus disease 2019 (COVID-19) patients. Ophthalmology.

[5] Xia J, Tong J, Liu M (2020). Evaluation of coronavirus in tears and conjunctival secretions of patients with SARS-CoV-2 infection. J Med Virol.

[6] Zhou Y, Duan C, Zeng Y (2020). Ocular findings and proportion with conjunctival SARS-COV-2 in COVID-19 patients. Ophthalmology.

[7] Siedlecki J, Brantl V, Schworm B (2020). COVID-19: ophthalmological aspects of the SARS-CoV 2 global pandemic. Klin Monatsbl Augenheilkd.

[8] Gaba WH, Ahmed D, Al Nuaimi RK (2020). Bilateral central retinal vein occlusion in a 40-year-old man with severe Coronavirus disease 2019 (COVID-19) pneumonia. Am J Case Rep.

[9] Finn AP, Khurana RN, Chang LK (2021). Hemi-retinal vein occlusion in a young patient with COVID-19. Am J Ophthalmol Case Rep.

[10] Yahalomi T, Pikkel J, Arnon R (2020). Central retinal vein occlusion in a young healthy COVID-19 patient: a case report. Am J Ophthalmol Case Rep.

[11] Varga Z, Flammer AJ, Steiger P (2020). Endothelial cell infection and endotheliitis in COVID-19. Lancet.

[12] Wong TY, Klein R, Klein BE (2001). Retinal microvascular abnormalities and their relationship with hypertension, cardiovascular disease, and mortality. Surv Ophthalmol.

[13] Casagrande M, Fitzek A, Püschel K (2020). Detection of SARS-CoV-2 in human retinal biopsies of deceased COVID-19 patients. Ocul Immunol Inflamm.

[14] Invernizzi A, Torre A, Parrulli S (2020). Retinal findings in patients with COVID-19: results from the SERPICO-19 study. EClinicalMedicine.

[15] Pirraglia MP, Ceccarelli G, Cerini A (2020). Retinal involvement and ocular findings in COVID-19 pneumonia patients. Sci Rep.

[16] Zou L, Ruan F, Huang M (2020). SARS-CoV-2 viral load in upper respiratory specimens of infected patients. N Engl J Med.

[17] Marinho PM, Marcos AAA, Romano AC (2020). Retinal findings in patients with COVID-19. Lancet.

[18] Walinjkar JA, Makhija SC, Sharma HR (2020). Central retinal vein occlusion with COVID-19 infection as the presumptive etiology. Indian J Ophthalmol.

[19] Montesel A, Bucolo C, Mouvet V (2020). Case report: central retinal artery occlusion in a COVID-19 patient. Front Pharmacol.

[20] Acharya S, Diamond M, Anwar S (2020). Unique case of central retinal artery occlusion secondary to COVID-19 disease. IDCases.

[21] Siddiqi HK, Libby P, Ridker PM (2021). COVID-19—a vascular disease. Trends Cardiovasc Med.

[22] Wu P, Duan F, Luo C (2020). Characteristics of ocular findings of patients with Coronavirus disease 2019 (COVID-19) in Hubei province, China. JAMA Ophthalmol.

[23] RKI (2021) COVID-19 Germany. https://experience.arcgis.com/experience/478220a4c454480e823b17327b2bf1d4. Zugegriffen: 20. Jan. 2021

[24] Aleem A, Nadeem AJ (2021). Coronavirus (COVID-19) vaccine-induced immune thrombotic thrombocytopenia (VITT).

[25] Yan R, Zhang Y, Li Y (2020). Structural basis for the recognition of SARS-CoV-2 by full-length human ACE2. Science.

[26] Lange C, Wolf J, Auw-Haedrich C (2020). Expression of the COVID-19 receptor ACE2 in the human conjunctiva. J Med Virol.

[27] Napoli PE, Nioi M, d’Aloja E (2020). The ocular surface and the Coronavirus disease 2019: does a dual „ocular route“ exist?. J Clin Med.

[28] de Figueiredo CS, Raony Í, Giestal-de-Araujo E (2020). SARS-CoV-2 targeting the retina: host-virus interaction and possible mechanisms of viral tropism. Ocul Immunol Inflamm.

[29] Anderson EL, Turnham P, Griffin JR (2020). Consideration of the aerosol transmission for COVID-19 and public health. Risk Anal.

